# Pharmacological inhibition of the CCL2-CCR2 axis fails to reduce inflammation in a rat model of acute lung injury

**DOI:** 10.1038/s41598-025-11971-2

**Published:** 2025-08-26

**Authors:** Marta Camprubí-Rimblas, Neus Tantinyà, Antonio Artigas, Raquel Guillamat-Prats

**Affiliations:** 1 Critical Care Research Center, Parc Taulí Hospital Universitari, Institut d’Investigació I Innovació Parc Taulí (I3PT-CERCA), Universitat Autònoma de Barcelona, Sabadell, Spain; 2 CIBER de Enfermedades Respiratorias (CIBERES), Sabadell, Spain; 3 Servei de Medicina Intensiva - Corporació Sanitària I Universitària Parc Taulí, Sabadell, Spain; 4 Germans Trias I Pujol Research Institute (IGTP), Badalona, Spain; 5 The Institute for Cardiovascular Prevention (IPEK), LMU University Hospital, LMU Munich, Pettenkoferstraße 9, 80336 Munich, Germany

**Keywords:** Acute lung injury, Macrophages, Monocytes, Cell recruitment, Acute respiratory distress syndrome, Inflammation, Acute inflammation, Preclinical research

## Abstract

New therapeutic approaches are needed to regulate inflammation and control monocyte recruitment in acute respiratory distress syndrome (ARDS). Excessive monocyte influx into the alveolar space can exacerbate lung damage, worsening patient outcomes. Delaying or reducing monocyte recruitment into the alveoli space after the injury has been proposed as a strategy to balance the inflammatory response and mitigate lung damage. In the present study, we assessed the possible role of the CCL2-CCR2 axis as a therapy for controlling acute lung injury after the initial neutrophil-driven influx. We administered a CCL2-antibody (CCL2-Ab) or a CCR2-antagonist (CCR2-Ant) locally into the lung following lung injury induced by HCl/LPS instillation. Our results show that after 24 h, both treatments transiently reduced monocyte infiltration into the bronchoalveolar space. After 72 h, neither CCL2-Ab nor CCR2-Ant sustained a reduced monocyte infiltration or significantly alleviated alveolar or lung inflammation. CCR2-Ant prevented an increase of alveolar permeability, but neither of both treatments, CCL2-Ab nor CCR2-Ant, improved lung damage or function. Our findings indicate that blocking the CCL2-CCR2 axis to control monocyte trafficking at early stages of lung injury is insufficient to control inflammation or prevent disease progression. These results highlight the complexity of ARDS pathophysiology and suggest that alternative strategies may be required to effectively modulate monocyte-driven lung inflammation.

## Introduction

Acute Respiratory Distress Syndrome (ARDS) is a pathology that affects the people’s lungs of all range of ages, from babies to the elderly^1–3^. Worldwide it is estimated that 2.2 million adults will be diagnosed with ARDS each year^4,5^. Patients with ARDS show a very high risk of mortality, with 30–35% dying due to ARDS and its associated complications^2,4^. Therefore, new therapeutic strategies are sought for regulating inflammation, leukocytes recruitment in the context of treatment of ARDS.

The recruitment of leukocytes is mainly mediated by chemokines (or chemotactic cytokines)-a family of small proteins-that bind to their corresponding seven-transmembrane domain G protein–coupled receptors expressed on the surface of leukocytes^6,7^. Chemokines and their receptors not only regulate leukocyte recruitment they also play a role in the activation, proliferation, and differentiation of the leukocytes^8^.

At early stages after lung damage, granulocytes are recruited into the alveolar space, followed by an increase of recruited monocytes that will convert into macrophages^1^. Among the chemokines involved, CCL2 (formerly called monocyte chemoattractant protein 1, MCP-1) plays a central role in the recruitment of classical (inflammatory) monocytes via its chemokine receptor 2 (CCR2)^9–12^; CCL2 is important in macrophages activation and trafficking^13,14^, and is predominantly produced by stromal cells, including fibroblasts, endothelial cells, and smooth muscle cells, among others^14,15^. CCL2 preferentially binds to CCR2, expressed in a wide range of organs and tissues including blood, brain, heart, kidney, liver, lung, ovary, pancreas, spinal cord, spleen, and thymus at different levels. CCR2 is highly expressed on circulating monocytes and tissue macrophage precursors. The CCL2-CCR2 axis governs the mobilization of monocytes from the bone marrow and spleen and directs their trafficking toward sites of tissue injury^16,17^.

Monocytes are recruited into the injured lung via the chemokine receptor CCR2 and its ligand CCL2^18,19^. In the context of ARDS patients, elevated levels of CCL2 in the bronchoalveolar lavage (BAL) have been related to worse lung injury, disease severity, continuous inflammation, and increased mortality^20–22^. Furthermore, CCL2-CCR2 axis and macrophages trafficking are implicated in the pathogenesis of several diseases^23–35^. These findings suggest that the CCL2-CCR2 pathway is a potential target for the treatment of inflammatory lung pathologies such as ARDS.

Pharmacological antagonism of CCR2 has been evaluated in several pre-clinical and clinical trials for treating autoimmune disease, cancer, atherosclerosis, and metabolic diseases to trigger the depletion of monocytes/macrophages, and with some success in ameliorating inflammation and monocyte-mediated injury^36–41^. Also, CCL2-neutralizing antibodies have been used in several diseases and have shown promising therapeutic efficacy^39,41^. Despite the growing interest in targeting this pathway, significant gaps remain in our understanding of its role during the acute phase of lung injury. Besides, they are not widely used due to the price and technical issues^42–45^.

In ARDS, the excessive monocyte infiltration into the alveolar space may have detrimental effect on the lung damage, worsening ARDS patients’ clinical outcome. In the present study, we aimed to investigate whether post-injury pharmacological inhibition of the CCL2-CCR2 axis could mitigate inflammation and lung damage in a well-established rat model of acute lung injury (ALI). By targeting monocyte recruitment after the initial neutrophil-driven phase, we sought to evaluate the therapeutic potential and limitations of this approach within a clinically relevant time frame.

## Methods

### Animals

Male Sprague–Dawley rats (Charles River, France) (200–250 g) were used in accordance with the European Community Directive 86/609/EEC and Spanish guidelines for experimental animals. The experimental protocol was approved by the institutional ethics committee of the Autonomous University of Barcelona (UAB) and the animal experimentation committee of Generalitat de Catalunya (approval number 11085). All the experiments were performed according the ARRIVE guidelines (Animal Research: Reporting of In Vivo Experiments). Authors confirm that all experiments were performed in accordance with relevant guidelines and regulations.

### ALI model

We used a double-hit model to induce ALI by using an intratracheal instillation of 300 µl of HCl (0.1 M at pH = 1.4) followed 2 h later by intratracheal instillation of LPS from Escherichia coli 055:B5 (30 µg/g of body weight) dissolved in 500 µl of saline^46^. This procedure was performed under sevoflurane anesthesia. Control animals received both intratracheal instillation with the same volume of saline.

### Treatment with CCL2-antibody or CCR2-antagonist

Nine hours after HCl administration, for injured animals, or saline, for control animals, the corresponding animals were treated with an intratracheal instillation under sevofluorane anesthesia of 500 µl of purified anti-human/rat/mouse MCP-1 (CCL2) antibody (0.16 µg/g body weight, Biolegend, (Reference: 505901) USA) or CCR2-antagonist (1.6 µg/g body weight, Tocris (Reference: 3129) Madrid).

### Experimental groups

The animals were randomly distributed into six experimental groups:

- Control (C): Saline instillation at 0 h, 2 h and 9 h.
- Control + CCL2-antibody (C + CCL2-Ab): Saline instillation at 0 h, 2 h and CCL2-antibody at 9 h.
- Control + CCR2-antagonist (C + CCR2-Ant): Saline instillation at 0 h, 2 h and CCR2-antagonist at 9 h.
- HCl and LPS (HCl/LPS): HCl instillation at 0 h and LPS administration at 2 h and saline at 9 h.
- HCL/LPS + CCL2-antibody (HCl/LPS + CCL2-Ab): instillation as in HCl + LPS and instillation of CCL2-antibody at 9 h.
- HCL + LPS + CCR2-antagonist (HCl/LPS + CCR2-Ant): instillation as in HCl + LPS and instillation of CCR2-antagonist at 9 h.

Rats were weighed and supervised throughout all the procedure, a n = 6 per group for the acute (24 h) experimental set up and a n = 12 animals per group for the long term (72 h) experimental set up were randomized, but the animal number of each analysis is indicated in the figure legends. Animals were sacrificed at 24 h or at 72 h by an intraperitoneal ketamine (90 mg/kg)-xylazin (10 mg/kg) administration and exsanguinated from the abdominal aorta. Collected blood was used for gases analysis (EPOC Blood Analysis System, Alere Healthcare) and then centrifuged at 3000×*g* to obtain the plasma that was aliquot and kept at − 80 °C for future analysis. The thorax was immediately opened, and the trachea and lungs were removed and weighed. Then, the left hilum was tied with a suture, and the right lung was removed, cut in pieces, and frozen. The left lung was gently lavage with five separated 5 ml aliquots of 0.9% NaCl to obtain the BAL samples. In another series of experiments, the lungs were fixed by intratracheal instillation of 4% paraformaldehyde (PFA), immersed in PFA for 24 h, and embedded in paraffin for histological analysis. The two femurs were obtained; one was used for flow cytometry analysis and the other was centrifuged to obtain the bone marrow cells from and then the cell pellet was resuspended in 1 ml of sterile PBS and centrifuged again. The lavage of the bone marrow cells was aliquoted in a new tube (BM-lavage) and was conserved at − 80 °C.

### Analysis of bronchoalveolar lavage fluid

Cell-free BAL supernatant was obtained from centrifuged BAL at 800×*g* for 10 min and the total protein concentration was determined by the bicinchoninic acid method (Pierce; Thermo Scientific; Rockford, IL, USA).

### Histology and immunostaining studies

For histological studies, unilobular lung were cut into 4 μm thick sections. The lung sections were stained with hematoxylin–eosin (H&E) and evaluated under light microscopy using a Nikon Eclipse Ti microscope and ImageJ software (ImageJ 1.40 g; W. Rasband, NIH, USA).

### Lung injury scoring (LIS)

The lung injury score (LIS) was quantified by two investigators blinded to the treatment groups. As shown in Table 1, the LIS was obtained by summing the scores for five variables (hemorrhage, peribronchial infiltration, interstitial edema, pneumocyte hyperplasia, and intraalveolar infiltration). A semiquantification of the immune cell infiltration into the lung tissue was also performed by the sum of intralveolar and peribronchial infiltration sub-scores. Scores were normalized to the number of fields evaluated. The resulting injury score was a value between zero and ten (both inclusive).

**Table 1:** Lung injury scoring system.

Parameter	Score per field
Hemorrhage	0–1
Peribronchial infiltration	0–1
Interstitial edema	0–2
Pneumocyte hyperplasia	0–3
Intraalveolar infiltration	0–3

### ELISA protein measurement

CCL2 was quantified by ELISA (R&D) in BM-lavage, plasma and BAL at the early (24 h) and late time point (72 h) following the protocol provided by the manufacturer. The amount of CCL2 is expressed by the picograms of CCL2 protein measured per ml in the three different samples, BAL, BM lavage and plasma. IL1β and IL6 were measured in the BAL at the early (24 h) and later time point (72 h) to assess the inflammatory burden; the amount of both interleukins is expressed by the picograms measured per ml of BAL.

### RNA extraction and real time q-PCR

RNA from lung tissue homogenate was extracted using chloroform-isopropanol isolation and purity was measured with spectrophotometer ND-1000 (Nanodrop, Thermo Fisher Scientific). RNA was retro-transcribed to cDNA using a kit (Takara Bio; Otsu, Japan), and real-time q-PCR was performed with SYBRgreen and the primers of interest (Table 2). GAPDH were used as housekeeping gene; the ΔΔCt method was used for correction.

**Table 2:** List of primers used in the qPCR analysis.

Gene	Forward primer	Reverse primer
GAPDH	5ʹ CTGTGCTTTCCGCTGTTTTC 3ʹ	5ʹ TGTGCTGTGCTTATGGTCTCA 3ʹ
IL-1β	5ʹ AAAAATGCCTCGTGCTGTCT C 3ʹ	5ʹ TCGTTGCTTGTCTCTCCTTG 3ʹ
IL-6	5ʹ CTGCTCTGGTCTTCTGGAGT 3ʹ	5ʹ GGTCTTGGTCCTTAGCCACT 3ʹ
CXCL1	5ʹ CCACACTCAAGAATGGTCGC 3ʹ	5ʹ GTTGTCAGAAGCCAGCGTTC 3ʹ
CXCL3	5ʹ TGCCTGAAGACCCTACCAAG 3ʹ	5ʹ GGGATCGACTCGGACGTTAT 3ʹ
CCR1	5ʹ CCAATCAGTGTGAGCAGAGC 3ʹ	5ʹ AGAGAAGAAGGGCAGCCATT 3ʹ
CCR5	5ʹ TGAGAAGAAGAGGCACAGGG 3ʹ	5ʹ AGCAGTGTGTCATCCCAAGA 3ʹ
CCR8	5ʹ AGTCCTCAGCACCTCTCCTA 3ʹ	5ʹ CTCCTCAGTTTCTTGCAGGC 3ʹ
CXCR3	5ʹ GTCCTAACACACTCCAGGCT 3ʹ	5ʹ GGCATGGCTCAGTAAAGGTG 3ʹ

### Flow cytometry analysis

#### BAL

One ml of BAL was used for flow cytometry. Cells were treated with ammonium chloride potassium (ACK) buffer to lysate the erythrocytes. After washing and Fc blocking with CD16/CD32 antibody, the cells were stained with the antibody mix for 30 min at 4 °C in the dark. Then cells were washed and measured by a FACSCantoII for different cell leukocyte subsets counts measurement and classification. Cell counts are expressed by ml of BAL. Data was analyzed by using FlowJo.

#### Blood

A total of 100 µL blood was incubated with 5 mL of ACK solution for erythrocytes lysis for 15 min at RT. After washing and Fc blocking with CD16/CD32 antibody, cells were stained with the antibody mix (antibodies indicated in Table 3) for 30 min at 4 °C in the dark. Then cells were washed and measured by a BD FACSCantoII (BD Biosciences) for different cell leukocyte subsets measurement and classification and cell counts are expressed by ml of blood. Data were analyzed by using FlowJo software.

**Table 3:** List of conjugated antibodies used in the flow cytometry analysis.

Antibody	Fluororchrome	Source	Clone	Dilution
CD45	Pacific Blue	BioLegend	OX-1	1:200
CD11b	PerCP-Cy5.5	BioLegend	OX-42	1:200
CD43	PE	BioLegend	W3/13	1:100
CD161	APC	BioLegend	3.2.3	1:100
CD86	FITC	eBioscience	B7-2	1:200
CD192 (CCR2)	Pacific Blue	BioLegend	K036C2	1:20
His48	FITC	eBioscience	HIS48	1:200

#### Bone marrow

One femur was centrifuged for 1 min at 10,000×*g*. ACK buffer was used for erythrocytes lysis and then after washing and blocking with Fc with CD16/CD32 antibody for 5 min on RT, cells were stained with an antibody mix (antibodies indicated in Table 3) for leukocytes subset classification for 30 min at 4 °C in the dark. After washing, cells were measured using a FACSCantoII for different cell leukocyte subsets classification and cell counts are expressed by femur. FlowJo software was used to analyze the data.

Myeloid cell subset populations were gated after selecting singlets and discarding lymphoid cells (CD45+ CD11b−); total myeloid cells: CD45^+^ CD11b^+^; monocytes: CD45^+^ CD11b^+^ CD43^high^ His48^low^ CD161^+^; Classical monocytes: CD45 + CD11b + CD43low His48high; Neutrophils: CD45 + CD11b + CD43high CD161− His48high CD86-. The gating strategy has been included in the Supplementary Fig. 1, and was following the Barnett-Vanes et al. strategy^47^.

### Statistical analysis of results

The results of the quantitative variables are expressed as mean ± SEM with 95% confidence intervals (CI). For group comparisons, one-way ANOVA followed by Tukey’s multiple comparison test was initially applied under the assumption of normal distribution. Given the limited sample sizes, particularly in the 24-h groups (n = 6 per group) and 72-h groups (n = 12 per group), we performed Shapiro–Wilk and D’Agostino-Pearson tests to evaluate the normality and log-normality of each dataset prior to applying parametric tests. In cases where normality could not be confirmed, we verified our findings using non-parametric alternatives (Kruskal–Wallis test followed by Dunn’s post hoc test). Both parametric and non-parametric analyses yielded comparable results, supporting the robustness of our conclusions. The outcomes of the normality assessments are provided in the Figure legends. We performed all the analysis using GraphPad Software Inc, USA. Values of p < 0.05 were considered significant.

## Results

In this study, we used a therapeutic strategy by blocking the CCL2-CCR2 axis using an intratracheal instillation of an antagonist of CCR2 (CCR2-Ant) or of a blocking antibody against CCL2 (CCL2-Ab) for treating ALI previously triggered by a double-hit model induced by HCl/LPS.

### Monocyte recruitment is temporarily reduced by CCL2-CCR2 blockade at early time-points

**Fig. 1:**
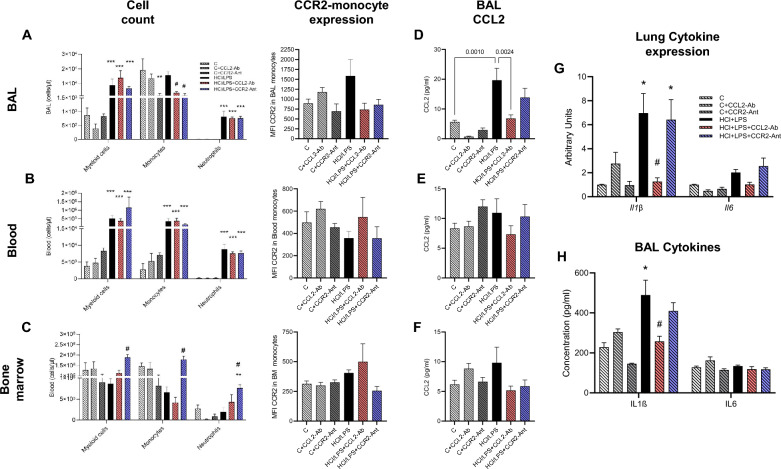
Cell counts of myeloid cells, monocytes and neutrophils, and mean intensity fluorescence (MFI, expression) of CCR2 in the surface of the monocytes measured by flow cytometry 24 h after HCl administration in the (**A**) bronchoalveolar lavage (BAL), (**B**) circulation (blood) and (**C**) in the femur’s bone marrow. CCL2 concentration 24 h after HCl administration in (**D**) BAL, (**E**) plasma, and (**F**) bone marrow lavage (BM-lavage). (**G**) Proinflammatory mRNA gene expression measured by real time qPCR in lung tissue homogenates. (**H**) IL1β and IL6 concentration was measured in bronchoalveolar lavage (BAL). Data shown by mean ± SEM. Exact p values or *p ≤ 0.05, **p ≤ 0.01, ***p ≤ 0.001 vs the control group; ^#^p ≤ 0.05 vs the HCl/LPS group are shown in the graphs to the respective compared group (n = 5–6 per group). After testing data for normality, one-way-ANOVA followed by Neuman–Keuls post hoc test or In Kruskal–Wallis test followed by Dunn’s post hoc test was performed.

We evaluated the effectivity of our treatments, CCR2-antagonist or CCL2-antibody, 24 h after the administration of HCl or saline. CCL2-CCR2 axis is mainly involved in monocytes recruitment, egressing bone marrow to the circulation and, travel to the site of the damage, in this occasion, the lung. Both treatments were able to reduce the number of monocytes in the BAL after 24 h of the insult (Fig. 1A). No changes were observed in the number of circulating monocytes at this timepoint (Fig. 1B). Neutrophil numbers were increased in BAL and circulation in all ALI groups, with no changes among treatments (Fig. 1A,B). This observation was accompanied by the fact that the CCR2-antagonist administration provoked a retention of monocytes in the bone marrow (Fig. 1C). We evaluated CCR2 expression by mean intensity fluorescence-MFI in the surface of monocytes and could assess that CCL2-antibody decreased substantially the expression of these receptor in the surface of BAL monocytes and CCR2-antagonist in the surface of BAL and BM monocytes, but any of those observations were significant (Fig. 1A–C). The administration of CCL2-antibody was able to reduce significantly CCL2 concentrations in BAL of injured animals after 24 h of the HCl/LPS insult (Fig. 1D), showing a clear effect of the CCL2-antibody. CCL2 concentration was not modified systemically in plasma or locally in the bone marrow (Fig. 1E–G).

The gene expression of *Il1β* and *Il6* was determined in lung homogenates, showing that CCL2-antibody administration was significantly attenuating the *Il1β* increase triggered by HCl/LPS (Fig. 1G); *Il6* was regulated similarly by in a low manner, so the observed changes did not reach any significance. In BAL, IL1β was raising up in all ALI groups, and it was controlled by the CCL2-antibody treatment; IL6 concentration in BAL was not altered (Fig. 1H).

### Limited effects of CCL2-CCR2 blockade on lung damage and function at later time-points

**Fig. 2:**
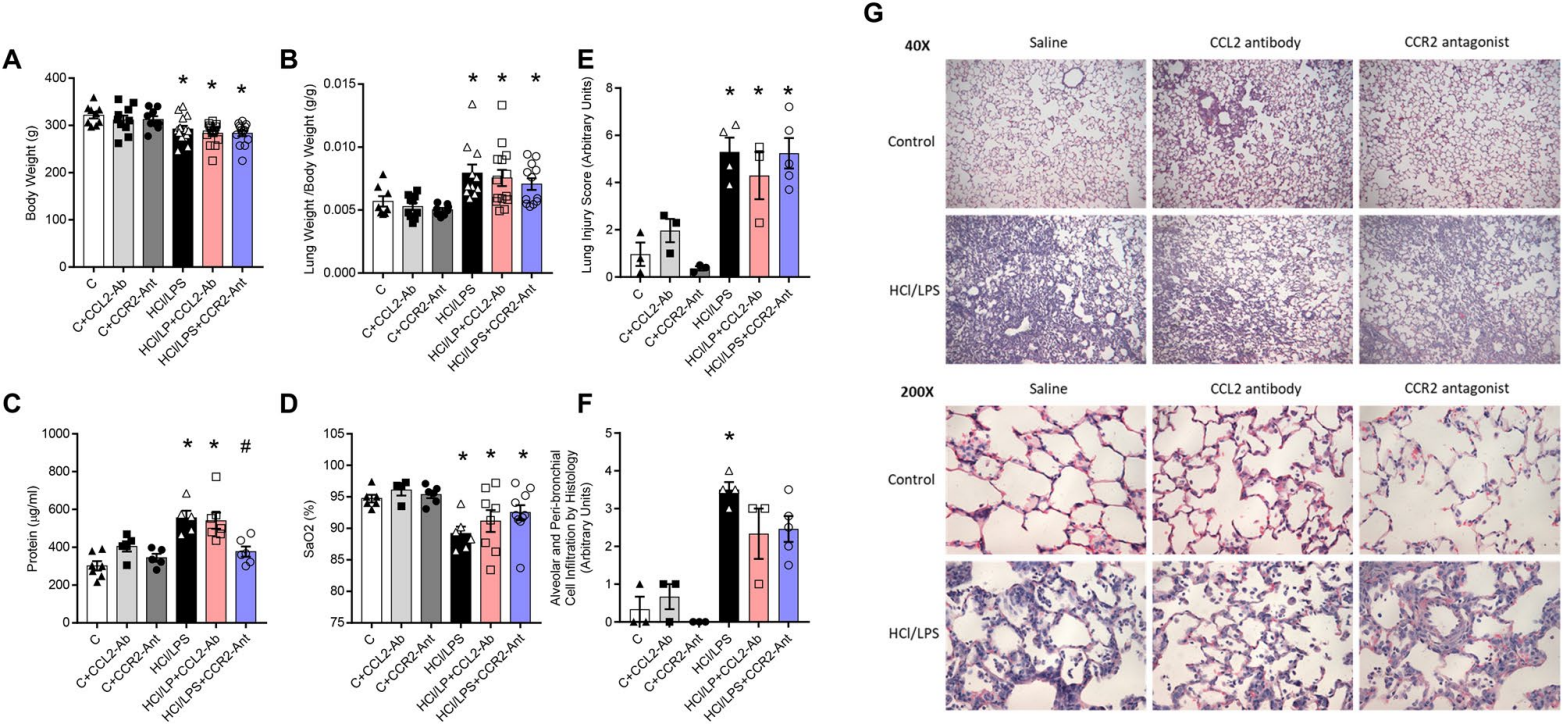
(**A**) Body weight of the animals after 72 h of the first instillation. (**B**) Lung weight of the animals corrected by their body weight (g/g). (**C**) Total protein measured in the bronchoalveolar lavage. (**D**) Oxygen saturation measured in aortic blood. (**E**) Lung injury scored where we measured hemorrhage, peribronchial infiltration, interstitial edema, pneumocyte hyperplasia, and intraalveolar infiltration. (**F**) Alveolar and Peri-bronchial Cell Infiltration lung histological sub-score. (**G**) Representative hematoxylin and eosin histological images at × 40 and × 200 magnification to have an overview and alveolar details for each experimental groups. N numbers are shown in each analysis, individual values are shown in all graphs. Data shown by mean ± SEM. *p ≤ 0.05 vs the control group; ^#^p ≤ 0.05 vs the HCl/LPS group. One-way-ANOVA followed by Neuman-Keuls post hoc test was applied.

Then we evaluated the effectivity of both treatments 72 h after HCl or saline administration to determine the beneficial role of CCL2-CCR2 axis for controlling ALI at later time points. The HCl/LPS instillation resulted in a loss of body weight and increased lung weight of the animals 72 h after HCl instillation (Fig. 2A,B). CCR2-antagonist and CCL2-antibody did not recover body weight nor reduce lung weight of the animals injured by the LPS.

HCl/LPS triggered a significant increase in the protein concentration suggesting damage in the alveolar epithelial barrier and higher permeability, as expected, and previously described in this model (Fig. 2C; p = 0.05)^46^. The treatment with CCL2-antibody could not prevent an increase in permeability; but, the CCR2-antagonist prevented a statistically significant increase in protein leakage into the BAL, suggesting a preserved alveolar epithelium due to the treatment (Fig. 2C; p = 0.05).

Once we measured oxygen saturation (SaO2) in blood, the decrease due to the HCl/LPS-triggered damage was not restored by any of both treatments (Fig. 2D). Both elevated considerably, but not significantly, the SaO2 levels compared to the HCl/LPS group (Fig. 2D).

The lung damage was evaluated histologically by a lung injury score (LIS) (Table 1); both treatments were able to reduce the HCl/LPS-induced lung injury; besides, we used two evaluated parameters of the LIS related to cell infiltration, to verify that both treatments slightly reduced alveolar and peribronchial cell infiltration without reaching significance (Fig. 2E,F). The histological images revealed that CCL2-antibody or CCR2-antagonist administration was not harmful as noticed in the control groups, but neither treatment effectively halted disease progression (Fig. 2G).

#### Impact on myeloid cell populations in BAL, blood, and bone marrow

**Fig. 3:**
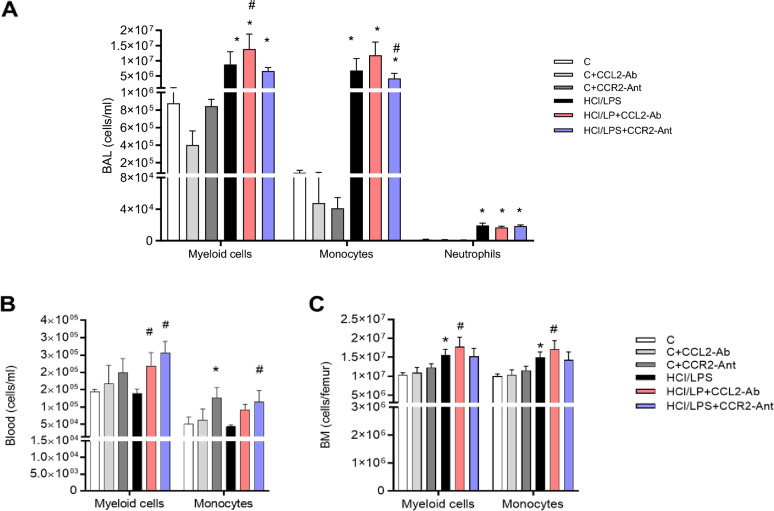
(**A**) Cell counts of total myeloid cells, monocytes and neutrophils measured in the bronchoalveolar lavage (BAL) by flow cytometry after 72 h of the HCl/LPS insult. (**B**) Myeloid and monocyte cell counts in the circulation. (**C**) Myeloid and monocyte cell counts in the bone marrow. Data shown by mean ± SEM. *p ≤ 0.05 vs the control group; ^#^p ≤ 0.05 vs the HCl/LPS group (n = 5–8 per group). After testing data for normality, one-way-ANOVA followed by Neuman–Keuls post hoc test or In Kruskal–Wallis test followed by Dunn’s post hoc test was performed.

We subsequently determined the myeloid cell numbers by flow cytometry in the BAL at 72 h. The analysis showed a diminished number of total myeloid cells, mainly macrophages, in healthy animals compared to the control group due to CCL2-antibody treatment (Fig. 3A). HCl/LPS instillation increased the number of myeloid cells (neutrophils, monocytes, and macrophages) in the BAL (Fig. 3A). CCL2-antibody did not modify the myeloid cell recruitment into the alveolar compartment, but the CCR2-antagonist slightly reduced the monocytes and classical monocytes numbers in BAL compared to HCl/LPS group (Fig. 3A). In blood, CCR2-antagonist treatment triggered the monocyte egress into circulation in healthy and injured rats. Increased myeloid cells were determined in the blood of the HCl/LPS animals that received CCL2-antibody or CCR2-antagonist (Fig. 3B).

In the bone marrow, HCl/LPS injured animals showed an increased number of myeloid and monocyte cell numbers compared to control animals (Fig. 3C). In addition, the HCl/LPS group treated with CCL2-antibody showed a significant increase of monocytes into the bone marrow, suggesting that the CCL2-antibody administration into the lung reduced or delayed the recruitment of monocytes to egress the bone marrow to later stages, retaining and increasing monocyte numbers in the bone marrow compartment.

#### Inflammatory and chemokine responses to CCL2-CCR2 blockade

**Fig. 4:**
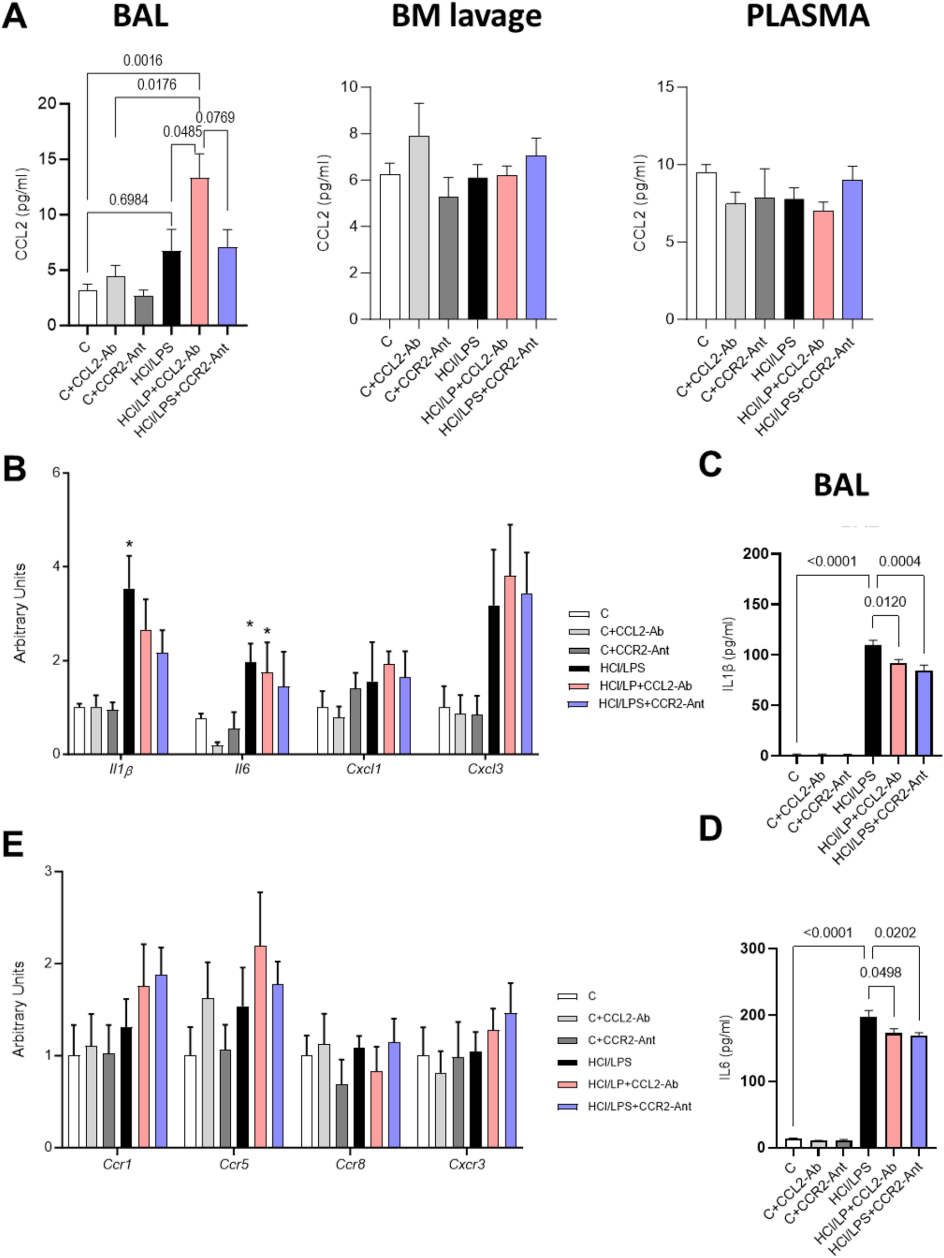
Expression of pro-inflammatory and recruitment chemokines in lung tissue after 72 h of the first insult. (**A**) CCL2 concentration in bronchoalveolar lavage (BAL), bone marrow lavage (BM-lavage) and plasma, (**B**) Proinflammatory and chemokine ligands mRNA gene expression measured by real time qPCR in lung tissue homogenates, (**C**) IL1β and (**D**) IL6 concentration in BAL. (**E**) Chemokine receptors mRNA gene expression measured by real time qPCR in lung tissue homogenates. Data shown by mean ± SEM. Exact p values or *p ≤ 0.05 vs the control group; ^#^p ≤ 0.05 vs the HCl/LPS group (n = 5–8 per group). After testing data for normality, one-way-ANOVA followed by Neuman–Keuls post hoc test or In Kruskal–Wallis test followed by Dunn’s post hoc test was performed.

To further explore the underlying mechanisms of the blockage of CCL2-CCR2 axis we measured CCL2 concentration in the BAL at 72 h. In the injured animals treated with CCL2-antibody, surprisingly, the alveolar CCL2 concentration was significantly increased compared to the control and injured animals, which correlates with the increased numbers of monocytes recruited in this compartment at this timepoint (Fig. 4A). However, no differences were determined in CCL2 concentration in the BAL in the injured animals treated with CCR2-antagonist compared to the HCl/LPS. No changes in CCL2 concentrations were observed in BM-lavage or plasma of any experimental group (Fig. 4A). The instillation of CCL2-antibody was not enough to maintain reduced alveolar CCL2 amounts after 3 days of ALI, suggesting a short effect of CCL2-antibody. The early effect of the CCL2-antibody administration found at 24 h was compensated delaying the CCL2 production and the mobilization of monocytes into the alveolar space, correlating with lung damage at later timepoints.

Focusing on the possible anti-inflammatory effects of our treatments, we evaluated in homogenates of lung the mRNA expression of classical pro-inflammatory cytokines *Il1β* and *I6*. HCl/LPS triggered their mRNA expression compared to the control group. CCR2-antagonist treatment did not modulate significantly mRNA expression of classical pro-inflammatory cytokines (*Il1β* and *Il6*) (Fig. 4B). CCL2-antibody slightly reduced *Il6* levels in healthy animals but did not have the same effect in the injured ones (Fig. 4B). We evaluated IL1β and IL6 protein concentration in the BAL, and both treatments reduced significantly the concentration of both pro-inflammatory interleukins compared to the HCl/LPS group (Fig. 4C,D). However, the IL1β and IL6 values were far away of the ones shown by a healthy animal, suggesting that CCL2-antibody and CCR-antagonist are only able to faintly reduce alveolar inflammation, with few positive consequences in the observed lung damage.

We also measured mRNA expression of *Cxcl1* and *Cxcl3* as main chemokines involved in myeloid cells recruitment into the lung together with CCL2. CXCL1 is binding to CXCR2 and plays a role in neutrophil trafficking, and CXCL3 is binding to CXCR2 and plays a major role in monocytes chemotaxis (Fig. 4B). *Cxcl1* expression was not modified by any treatment (Fig. 4B). *Cxcl3* expression augmented due to HCl/LPS instillation, but neither CCL2-antibody neither CCR2-antagonist modified *Cxcl3* expression in the lung (Fig. 4B). Our results suggest that the blockage of the CCR2-CCL2 axis does not trigger the up-regulation of any other main chemokine involved in monocyte recruitment into the lung as a compensatory mechanism.

To answer if any of both treatments were modifying other receptors involved in monocytes recruitment, we measured the expression of *Ccr1*, *Ccr5*, *Ccr8*, and *Cxcr3* in lung tissue. The measured receptors were neither modulated by CCL2-antibody nor by CCR2-antagonist (Fig. 4E).

## Discussion

The present study demonstrates that controlling monocyte trafficking by the pharmacological inhibition of CCL2-CCR2 axis at early stages of acute damage is ineffective for reducing completely inflammation or limiting the progress of lung damage. Our objective was to assess the efficacy of a CCL2-antibody or CCR2-antagonist treatment, mirroring the clinical scenario in ARDS patients upon hospital arrival. Unlike prior studies where these agents showed promise as preventive treatments, our results indicate that a single dose of either antibody or antagonist reduces monocyte recruitment in the alveolar space 24 h post HCl/LPS-induced lung injury. However, this reduction is not maintained on time, and 72 h later only rested a slightly decreased inflammation in lung tissue but not an improvement in lung damage or function.

Inflammation serves as the primary mechanism for monocyte and neutrophil recruitment into tissues. The acute phase of pulmonary inflammation is dominated by the early recruitment and activation of neutrophils, which play a central role in initiating tissue damage through the release of proteases, reactive oxygen species, and neutrophil extracellular traps (NETs). In our study, CCR2 or CCL2 inhibition was initiated after HCl/LPS administration, at a stage when substantial pulmonary injury may have already occurred. Given that CCR2 primarily regulates monocyte trafficking rather than neutrophil recruitment, its pharmacological blockade might be insufficient to counteract the acute, neutrophil-driven phase of lung injury. This could explain the limited efficacy observed in our model. Our findings underscore the importance of therapeutic timing and suggest that CCL2-CCR2 inhibition may be more relevant in later phases of inflammation or in modulating chronic sequelae, rather than as an intervention during the initial immune response. Further studies using earlier intervention timepoints or combination strategies targeting both neutrophil and monocyte pathways may be needed to more effectively modulate the course of ALI.

Monocytes, a complex population originating in the bone marrow, express various chemokine receptors, including CCR2, associated with inflammatory responses and infection defense. CCL2 is the main ligand for CCR2^10,48^. In non-inflammatory conditions, without any damage, the migration of myeloid cells-monocytes and neutrophils-from the bone marrow to circulation is maintained in a stable pool. The mechanisms regulating mobilization of monocytes from bone marrow to the site of damage, the lung, in response to infection and inflammation are not entirely well understood. Over the past years, it has been described that the CCL2-CCR2 axis plays a crucial role in regulating monocyte traffic from the peripheral circulation and the recruitment of monocytes to inflamed sites^16,49^. Blockade of CCR2 could induce monocytopenia^50^ and increase susceptibility to infection^16,51–54^, suggesting its potential as a therapeutic target. CCR2 deficient mice show reduced monocyte egress from the bone marrow and mobilization to injured organs, predicting that CCR2 blockade may be a therapeutic option to reduce inflammation associated with the recruited monocytes^11,49,55^. Therefore, the development of antagonists for CCR2 has called much interest. Small molecule antagonists for CCR2 like INCB3344 have shown efficacy in preclinical inflammatory models^56^. However, our study found that a single dose of CCR2-antagonist post-injury failed to inhibit long-term monocyte trafficking, explaining the lack of reduction in lung damage progression. In our study, the single dose of CCR2-antagonist after 9 h of inducing an ALI by HCl/LPS instillation, that is the time when the monocyte mobilization and infiltration have already started, reduced the infiltrated monocytes in the alveolar space 24 h after the insult, but this reduction was not persistent nor sustained at 72 h, indicating that a single dose of CCR2-antagonist is not sufficient to inhibit the long-term monocyte trafficking into the alveolar space. The CCR2-antagonist increased the number of monocytes in the bone marrow in injured animals at 24 h, and the circulating monocytes in healthy and injured animals at 72 h. We speculate that the blockage of CCR2 first retains monocytes in the bone marrow and then reduces the migration capacity of monocytes, enhancing that they remain in circulation; this can explain our observations in blood and also the faintly reduction of pro-inflammatory proteins in the BAL.

CCL2, produced in response to various inflammatory stimuli, is primarily secreted by monocytes and macrophages^57,58^. CCL2 is the target for pharmacological intervention in several studies: multiple sclerosis^59^, rheumatoid arthritis^60^, and atherosclerosis^61^. In vivo, CCL2-knockout is not lethal, but these animals show abnormalities in monocyte recruitment and cytokine expression^62^. Previous studies targeting CCL2 have shown promise in reducing local inflammation^63–65^. In a pneumonia model of intratracheal *Pseudomonas aeruginosa* intraperitoneal administration of anti-MCP1/CCL2 aggravated lung tissue injury and did not decrease infiltrated cells in the alveolar space 48 h after infection^66^. In our experimental set up, the administration of CCL2-antibody post-damage resulted in initial reduction of monocytes in the alveolar space, compensated by increased infiltration after 3 days, correlating with no improvement in lung damage or function. Augmented numbers of circulating monocytes and delayed mobilization due to CCL2-antibody administration may explain this phenomenon.

Interestingly, we observed a reduction in CCL2 expression following administration of the CCL2 inhibitor 24 h after injury. Although CCL2 inhibitors are primarily designed to neutralize ligand activity and prevent receptor binding, our results suggest additional regulatory effects on CCL2 levels. One plausible explanation is that the neutralizing antibody used in our study may have promoted the formation of immune complexes with free CCL2, facilitating its early clearance from the extracellular environment. Furthermore, inhibition of CCL2 signaling may have interrupted positive feedback loops involving CCR2 activation, which are known to drive further CCL2 expression in monocytes, macrophages, and pulmonary resident cells. This feedback suppression could result in reduced transcription and production of CCL2. Additionally, dampened recruitment and activation of CCL2-producing monocytes due to the inhibitor may have contributed to the overall decrease in pulmonary CCL2 levels. The sustained increase in CCL2 observed at 72 h may indicate a secondary or compensatory wave of chemokine production associated with ongoing monocyte/macrophage activation or early reparative responses. This pattern is consistent with studies linking CCL2 to both inflammation and tissue remodeling, suggesting that its role may extend beyond initial leukocyte recruitment during ALI. Together, these mechanisms may highlight a more complex interplay between ligand inhibition and chemokine expression dynamics in the injured lung.

Overall, blocking the CCL2-CCR2 axis with a single dose of antibody or antagonist failed to maintain reduced monocyte infiltration or translate into lung damage recovery. The challenge lies in the chemokine system’s redundancy and capacity to counter-regulate alternative pathways^67^. Despite modest, but significant, reductions in IL1β and IL6 levels at 72 h. Although no significant changes in lung architecture or SaO₂ were observed, this may reflect transient or compartmentalized regulatory mechanisms not captured at the analyzed time points or by bulk tissue analysis. Our study sheds light on the complexities of targeting chemokine-chemokine receptor interactions and their great redundancy and underscores the need for more comprehensive therapeutic strategies and management in the complex pathophysiology of ARDS^24^.

While CCR2-dependent monocyte emigration from the bone marrow is well-established, the involvement of other receptors like CCR1, CCR5, CCR8, or CXCR3, and chemokines involved in lung injury^68^, like CXCL1 and CXCL3, complicate therapeutic targeting^69^. Injured pulmonary cells and CCR2 monocytes are known to contribute to neutrophils infiltration in the alveolar space^70^. In our work, blocking the CCL2-CCR2 axis failed to modify other chemokines like CXCL1 and CXCL3, indicating a broader inflammatory response beyond CCL2-CCR2 interactions.

Monocytes are a functionally diverse population that contribute in both the induction and resolution of the ALI. While CCL2-CCR2 signaling primarily mediates the recruitment of classical monocytes, their fate within the lung is shaped by local cues that drive polarization toward either tissue-damaging or reparative phenotypes. Our study focused on the inhibition of this axis, but we recognize that monocytes interact with resident macrophages, neutrophils, and structural lung cells in a dynamic microenvironment. The limited therapeutic effect observed may reflect the complexity of monocyte functions and their integration into broader immune networks. Future studies exploring monocyte heterogeneity, fate, and interactions at single-cell resolution may better elucidate their contribution to lung injury and identify more precise therapeutic targets.

The complexity of ARDS pathophysiology lies in its multifactorial nature and the rapid, sequential activation of multiple immune cell populations and pathways, being mediated by a more complex inflammatory response, not only linked to the CCL2-CCR2 axis and monocyte infiltration. Disappointing outcomes with CCR2 antagonists in clinical settings underscore the necessity for alternative approaches^71^. Timing, site of administration, and model severity may contribute to discrepancies in pre-clinical studies. Future studies should explore sustained blockade or multi-targeted approaches to enhance efficacy while considering cost implications.

Our findings have several limitations that we could not manage to overcome. A sustained blockade of the CCL2-CCR2 axis (with a continuous administration of the treatments or multiple doses every 12/24 h) might be helpful to see an effect in the lung damage at 72 h. However, to rise the number of doses will increase the cost of the treatment making it unaffordable for the clinical use. A blockage of not only monocytes but also neutrophils might be more beneficial than only targeting monocytes. Also, targeting several chemokine-chemokine receptors axes at the same time could be more effective in reducing monocyte infiltration into the lung and possibly also inflammation. All these strategies may be further evaluated in future experimental studies.

Our findings suggest that transiently blocking CCR2 or CCL2 is insufficient to control inflammation in ALI. Targeting other immune cells like neutrophils or employing multi-targeted strategies, such as combining the blocking of CCL2-CCR2 axis with corticosteroids, may offer more promising outcomes. Therefore, while the CCL2-CCR2 axis blockade shows limited efficacy, alternative approaches warrant further investigation.

## Supplementary Information

Supplementary Figure 1.

Supplementary Legends.

## Data Availability

The published data are available and will be shared from the corresponding authors on request.
