# Supplementary figures and images for: Pharmacological inhibition of the CCL2-CCR2 axis fails to reduce inflammation in a rat model of acute lung injury
Source: Sci Rep. 2025 Aug 26;15:31368. doi: 10.1038/s41598-025-11971-2 (PMC12381131; doi:10.1038/s41598-025-11971-2)

Supplementary Figure 1

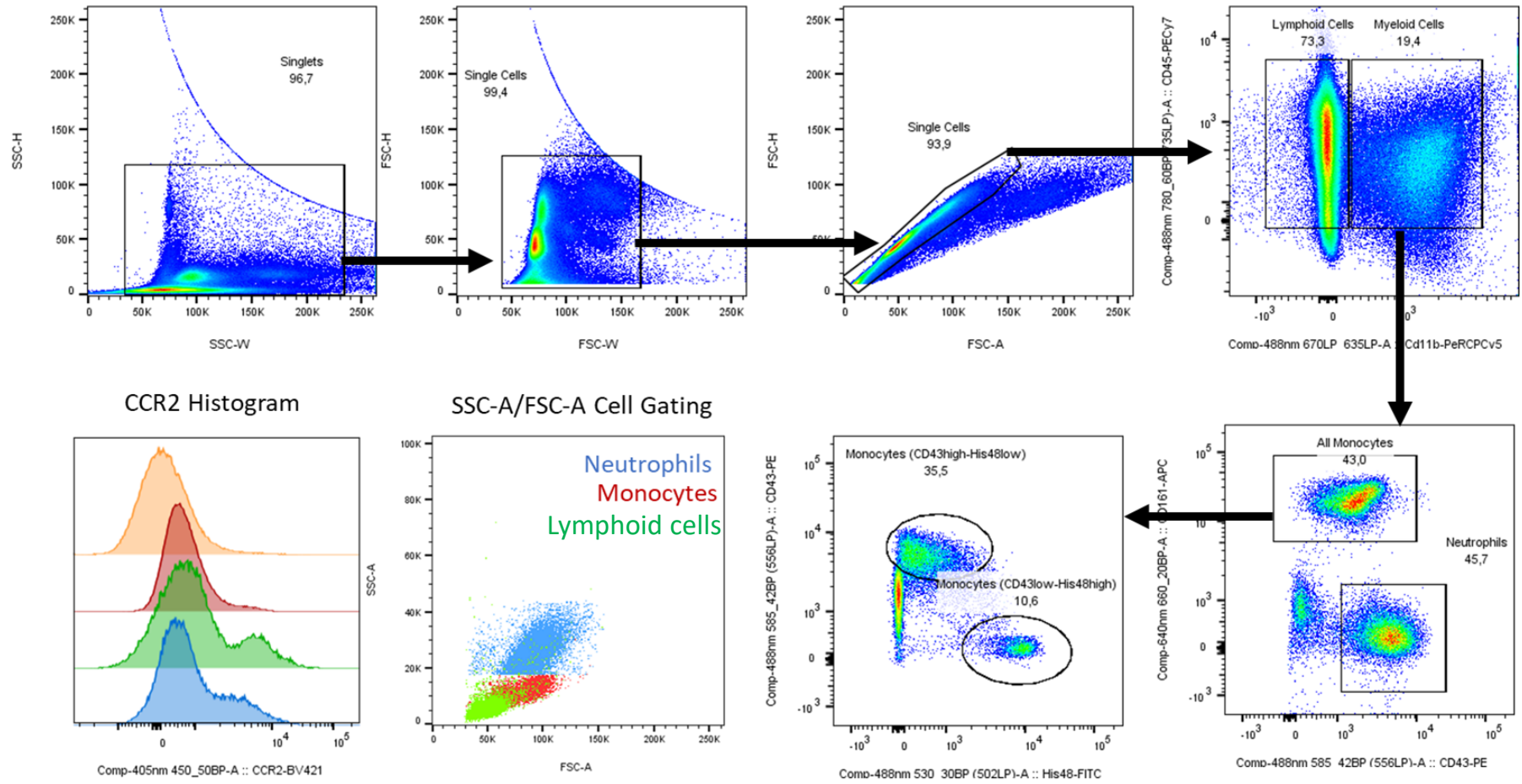

Supplement: Supplementary file 1 — Supplementary Figure 1. [file 41598_2025_11971_MOESM1_ESM.pdf]
