# Supplementary material for: Pharmacological inhibition of the CCL2-CCR2 axis fails to reduce inflammation in a rat model of acute lung injury
Source: Sci Rep. 2025 Aug 26;15:31368. doi: 10.1038/s41598-025-11971-2 (PMC12381131; doi:10.1038/s41598-025-11971-2)
Supplement: Supplementary file 2 — Supplementary Legends. [file 41598_2025_11971_MOESM2_ESM.docx]

**Supplementary Figure 1:** Flow Cytometry gating strategy used for the different myeloid cell populations. Gatting of lymphoid (CD45+CD11b-) and myeloid (CD45+CD11b+) cell populations, Monocytes (CD45+CD11b+CD161+, CD43+), Neutrophils (CD45+CD11+CD43high) and we sub-gated them to confirm His48 high expression and negative CD86 (not shown). Classical monocytes (CD45+CD11b+CD161+ CD43^high^ His48^low^) and not classical monocytes (CD45+CD11b+CD161+ CD43^low^ His48^high^). We show in a dot-plot the gated neutrophils, monocytes and lymphoid cells overlapping in different colors to confirm the SSC-A/FSC-A. Histogram of CCR2 present in the surface of monocytes in different groups and samples are shown to show the clear expression in some populations with increased mean fluorescence intensity.
